# Supplementation of Vitamin C Reduces Blood Glucose and Improves Glycosylated Hemoglobin in Type 2 Diabetes Mellitus: A Randomized, Double-Blind Study

**DOI:** 10.1155/2011/195271

**Published:** 2011-12-28

**Authors:** Ganesh N. Dakhale, Harshal V. Chaudhari, Meena Shrivastava

**Affiliations:** ^1^Department of Pharmacology, Indira Gandhi Government Medical College, Nagpur 440018, India; ^2^Department of Pharmacology, Shree Sathya Sai Medical College and Research Institute, Chennai 603108, India

## Abstract

No study has ever examined the effect of vitamin C with metformin on fasting (FBS) and postmeal blood glucose (PMBG) as well as glycosylated hemoglobin (HbA1c) in the treatment of type 2 diabetes mellitus (DM). The goal was to examine the effect of oral vitamin C with metformin on FBS, PMBG, HbA1c, and plasma ascorbic acid level (PAA) with type 2 DM. Seventy patients with type 2 DM participated in a prospective, double-blind, placebo-controlled, 12-week study. The patients with type 2 DM were divided randomly into placebo and vitamin C group of 35 each. Both groups received the treatment for twelve weeks. Decreased PAA levels were found in patients with type 2 diabetes mellitus. This level was reversed significantly after treatment with vitamin C along with metformin compared to placebo with metformin. FBS, PMBG, and HbA1c levels showed significant improvement after 12 weeks of treatment with vitamin C. In conclusion, oral supplementation of vitamin C with metformin reverses ascorbic acid levels, reduces FBS, PMBG, and improves HbA1c. Hence, both the drugs in combination may be used in the treatment of type 2 DM to maintain good glycemic control.

## 1. Introduction

Diabetes mellitus (DM) is one of the major metabolic disorders associated with great deal of morbidity and economic cost. Apart from hyperglycemia, DM is also characterized by oxidative stress, inflammation, and insulin resistance [1]. Several investigators have implicated the role of free radical-mediated pathology in diabetes mellitus [2, 3]. The illness has poor outcome in spite of the best currently available treatments. Hence, development of novel strategies to improve the outcome will be of great benefit. Presently available oral hypoglycemic agents do not show marked improvement in oxidative stress in diabetic patients [4]. Ascorbic acid (vitamin C), an antioxidant vitamin, plays an important role in protecting free radical-induced damage. Previous study has shown decrease in basal vitamin C level in type 2 DM [5]. Vitamin C is structurally similar to glucose and can replace it in many chemical reactions and thus is effective for prevention of nonenzymatic glycosylation of protein [6].

Up-to-date, available literature suggests conflicting results related to supplementation of vitamin C and improvement in blood glucose level and glycosylated hemoglobin (HbA1c) [7, 8]. However, no study has ever examined the effects of vitamin C with metformin in the treatment of type 2 diabetes mellitus, especially on postmeal blood glucose (PPBG) level. Recent study reported reduction in fasting blood glucose (FBG) and glycosylated hemoglobin after supplementation with vitamin C, but it was an open-label, noncontrolled, and short duration study [5]. Therefore, it was considered worthwhile to measure the effect of vitamin C along with metformin on levels of FBG, PPBG, HbA1c, and plasma ascorbic acid in a double-blind, controlled study for longer duration in patients with type 2 diabetes mellitus.

## 2. Materials and Methods

### 2.1. Patients, Inclusion, and Exclusion Criteria

Seventy patients with type 2 diabetes mellitus participated in a prospective, double-blind, placebo-controlled, noncrossover, 12-week study approved by Institutional Ethics Committee of Indira Gandhi Government Medical College, Nagpur, India. All patients gave their informed consent prior to their inclusion in the study. The inclusion criteria were patients from Outpatient Department (OPD) of Medicine and diagnosed patients of type 2 DM of age group between thirty to sixty years who were on metformin and having fasting blood glucose level in the range of 126 to 250 mg/dL. Exclusion criteria for patients were fasting blood glucose level more than 250 mg/dL, medical illnesses including other endocrine, metabolic, type 1 DM, pregnancy, isolated postprandial hyperglycemia, and age more than 60 years or less than 30 years. None of the subjects was a regular drinker, heavy smoker, or had been taking any psychotropic drug. Routine investigations like electrocardiogram, serum electrolytes, blood urea, serum certainties, and liver function test were performed to rule out active medical problems in all patients. Patients who have received vitamin C or any other antioxidant over the last three months were also excluded from the study. The period of the present study was from February 2009 to November 2010.

### 2.2. Sample Size, Randomisation, and Treatment

Patients with type 2 DM were divided randomly into two groups, A and B of 35 each. Sample size was calculated with standard deviation taken from the previous study and level of significance at 0.05 with power as 80%. Block randomization procedure was used for random allocation of study drugs, that is, vitamin C and placebo with blocks of size 4 in equal proportions to ensure uniform allocation ratio (1 : 1). The study being double-blind, the drugs were identical in formulation, shape, size, weight, texture, and packing. The randomized treatment allocation sequence was generated by statistician using random number table. It was handed over along with identical plastic containers filled with the study drugs (sixty each of vitamin C and placebo) to a third person not directly involved in this study. This person labelled the containers according to the random allocation sequence of patients with drugs provided. The code of this random allocation sequence was retained in the sealed envelope by this person and was opened only after the completion of study during analysis of the data. The patients as well as the investigators were unaware of the treatment (Vitamin C or placebo) being administered. Drug was issued to patients for duration of thirty days at a time. Patients were asked to bring the unused drugs and container during the followup. Ninety percent consumption was considered to be compliant. The return drugs were discarded. Drugs were decoded at the end of trial. Group A received vitamin C with metformin and group B received placebo along with metformin. The dose of vitamin C was 500 mg twice a day and decided on the basis of previous study. All patients received open label tablet metformin 500 mg twice daily orally with lunch and dinner, as it is unethical to give either only placebo or vitamin C to diabetes patients. These patients, who were on metformin, were not on any other medicine including other antidiabetic agents. The intake of metformin did not start at the same time as the intake of vitamin C because the patients were on metformin for variable period within 0 to 6 months but not more than 6 months.

 In our preliminary experiment, it was determined that subjects reached a new lower steady-state plasma concentration after one week on controlled diet. Placebo or vitamin C supplementation was started after one week of vitamin C restricted diet. Patients were given a new supply of tablets at the end of each fourth week. Same doses were maintained throughout the study. Patients were not stabilised before enrolment in the study as they were satisfying our main inclusion criteria. But if we find increased fasting blood sugar level beyond 250 during the study period, then we exclud that patient from the study. No comorbid condition or infection occurred to these study patients during the study period. After study period was over, subjects were handed over to respective physician.

### 2.3. Diet and Investigations

All patients were maintained on their usual dietary pattern while limiting their consumption of vitamin C-rich foods throughout the study. As patients were on self-selected diet, each patient was instructed by a dietician to use comprehensive food list that contained food items by type, portion size, method of preparation, and vitamin C content. This enabled patients to substitute foods with low-vitamin content for those patients who normally consume higher levels of vitamin C and also to ensure that their daily intake from dietary sources would remain the same. Compliance to dietary restrictions of ascorbic acid was determined by obtaining a 24 hr dietary recall from the subjects during each month period. Fasting, postmeal blood glucose, glycosylated hemoglobin, plasma ascorbic acid, and liver and kidney function tests were repeated after twelve weeks. Fasting blood samples were obtained at baseline for the assessment of fasting, glycosylated hemoglobin, and plasma ascorbic acid (one week after vitamin C-restricted diet), whereas postmeal blood samples were taken for assessment of postmeal blood glucose 2 hours after meal. General clinical safety was monitored by vigilant followup of patients for treatment of emergent adverse events, if any, and recorded in the case report form. Patients with adverse drug reaction were treated appropriately by the physician in medicine OPD. Plasma ascorbic acid was estimated by a single-step calorimetric method using modified acid phosphotungstate reagent [9]. Supernatant was used to measure absorbance at 700 nm. Standards of pure ascorbic acid obtained from Sigma Company, USA, in the range of 0.10 to 0.90 mg % were prepared in 0.5% oxalic acid solution. For all the investigations, chemicals used were of analytical reagent grade. Fasting as well as postprandial blood glucose level and HbA1c was quantitatively estimated by glucose oxidase method and cation-exchange resin method with the use of semiautoanalyser, TRANSASIA, ERBA, CHEM-5 PLUS, respectively.

### 2.4. Statistical Analysis

Results were expressed as Mean ± standard error of mean (SEM). Group differences were ascertained by either paired or unpaired *t-*test. Relationship between variables was determined by means of either Pearson's or Spearman's correlation coefficient depending on distribution of the data. Chi-square test was used for analysis of demographic data. Two-tailed *P* value was used throughout, and the *P* values less than 0.05 were adjudged statistically significant. GraphPad Prism version 5.00 software was used for analysis.

## 3. Results

The mean age of the patients with diabetes mellitus in vitamin C group (48.33 ± 1.39 years) and placebo group (45.88 ± 1.42 years) was not significantly different from each other (Table 1). Fasting, postmeal blood glucose, and plasma ascorbic acid levels did not differ among subjects before receiving placebo and vitamin C treatment (*P* > 0.05).

In placebo group, levels of fasting and postmeal blood glucose reduced significantly (*P* < 0.05), and at the same time, levels of plasma ascorbic acid increased after 12 weeks of treatment compared to pretreatment levels but was nonsignificant. Reduction in glycosylated hemoglobin was seen but it did not reach to statistically significant level (Table 2). In patients receiving vitamin C, reduction in fasting and postmeal blood glucose was significant at week twelve. In contrast, plasma ascorbic acid levels raised significantly after twelve weeks of treatment. Simultaneously after twelve weeks, significant reduction was observed in glycosylated hemoglobin (Table 3).

To test whether oral supplementation of vitamin C is better in reversing fasting, postmeal blood glucose, and glycosylated hemoglobin as compared to placebo, we compared effects of drugs in placebo and vitamin C group after 12 weeks of treatment, taking into consideration the change from baseline values of these parameters. Decrease in fasting and postmeal blood glucose level was significant after twelve weeks in vitamin C group compared to placebo group. In contrast, supplementation of vitamin C increased plasma ascorbic acid significantly in vitamin C group compared to placebo group. At the same time, glycosylated hemoglobin level decreased significantly in vitamin C group compared to placebo group (Table 4). No correlation existed between plasma ascorbic acid and any of the parameters such as FBS, PPBS, and HbA1c for both vitamin C and placebo group.

Of the seventy patients entered in the trial, four were withdrawn (two in placebo group and two in vitamin C group). The most common cause was failure to reattend; one patient was dropped out in placebo group because of uncontrolled blood glucose level at the end of four weeks and was shifted to the other drug. No serious adverse event was reported, and no abnormalities in laboratory test were found during trial period.

## 4. Discussion

### 4.1. Vitamin C and DM

The primary finding of the present study is a significant decrease in FBG, PPBG, and HbA1c and an increase in plasma ascorbic acid level after vitamin C supplementation along with metformin in patients of type 2 DM. To the best of our knowledge, this is the first report, related to significant improvement in all above parameters after concomitant use of metformin and vitamin C. The results of this study are in agreement with previously published data showing betterment in glycemic control with vitamin C supplementation [10, 11]. In our study, patients also received metformin and it is an established first-line drug for treatment of type 2 DM. Hence, it is difficult to say whether this beneficial effect of ascorbic acid supplement could be attributed to its effect on the underlying disease or correction of the inadequate vitamin C status. The exact mechanism by which vitamin C brings about these changes is not known. It is well documented that there is an increased production of damaging free radicals in type 2 DM patients. Glucose autooxidation, protein glycosylation, formation of advanced glycation end products, and polyol pathway are involved in generation of oxidative stress, implicated in the origin of both type 1 and 2 DM [12]. The protection against such damage can be offered by free radical-scavenging antioxidants.

Increased demand for vitamin C to compensate the increased oxidative stress and impaired transport or dietary deficiency of vitamin C may be contributing to decreased levels of plasma vitamin C levels observed in type 2 DM patients [13]. High but physiologic concentrations of ascorbic acid can directly inhibit erythrocyte aldose reductase and provide a rationale for the use of oral vitamin C supplements in diabetes [14]. A significant inverse relationship between plasma AA and DNA damage in type 2 DM patients indicates that poorly controlled diabetic subjects might benefit from increased dietary vitamin C [15]. Ascorbic acid supplementation for diabetic subjects may provide a simple means of preventing and ameliorating the complications of diabetes. The weak methodology in past research leads to conflicting results as the studies were not controlled. Therefore, like our trial, randomized, double-blind clinical trials of ascorbic acid supplementation for longer duration should be a high priority for establishment of role of ascorbic acid in diabetes.

Supplementation of vitamin C reduced FBG and HbA1c levels in type 2 DM patients; it may be related to supplementation of higher dose of vitamin C. In earlier study, supplementation with 500 mg/day vitamin C in diabetic patients resulted in no changes in FBG and HbA1c in comparison with placebo. This may be linked to low dose of vitamin C used in this study [16]. Supplementation of AA per day for two weeks significantly reduced erythrocyte sorbitol and red cell sorbitol: plasma glucose (S/PG) ratio, and it was concluded that 1,000 mg AA per day supplementation might provide a simple, safe, and effective means of preventing and ameliorating chronic complications of diabetes. But, paradoxically the fasting plasma glucose levels revealed no change. Shorter duration of study may be implicated for this finding. Higher dose of AA provided improved glycemic control among type 2 DM subjects and both FBG and HbA1c improved [8]. Another study reported that AA may improve glycemic control, lowering both FBG and HbA1c [17]. The improvement of glycemic control was mainly initiated by a beneficial effect of antioxidant on *β* cells. However, we cannot totally deny the possibility that the antioxidant treatment could have exerted an influence on target tissues other than the *β* cells such as muscle and fat. Antioxidant treatment has beneficial effects on preservation of *β* cell function in diabetes, although the effects may not be exerted totally through its direct action on *β* cells. Also, regardless of the influence on insulin sensitivity, the antioxidant treatment indeed reduced blood glucose levels. Hence, vitamin C reduced glucose toxicity and contributed in part to the prevention of a decrease of *β* cell mass and insulin content. Another explanation proposed for reduction of blood glucose level is that plasma vitamin C levels seem to play a role in the modulation of insulin action in diabetic subjects. Vitamin C-mediated increase in insulin action is mainly due to an improvement in nonoxidative glucose metabolism [18].

Second most important observation of the study was significant reduction in HbA1c at twelve weeks in vitamin C-supplemented group in comparison with placebo group. But unfortunately, we could not find significant correlation between plasma ascorbic acid and HbA1c at the baseline and at twelve weeks. A significant decrease was noted in serum HbA1c in patients supplemented with vitamin C for six weeks [19]. This could be attributed to competition of vitamin C with glucose for reaction with amino groups on the hemoglobin beta chain [20]. Further explanation proposed that the increase of serum antioxidant glutathione and the decrease of glycosylated hemoglobin after long-term ascorbic acid supplementation are related to each other [21]. Our results do not rule out metabolic or pharmacokinetic interactions between ascorbic acid and metformin. In either case, results of the present study point to ascorbic acid as critical in modulating biochemical effects of metformin. In previous studies, normal dietary vitamin C intake was studied in detail but found to be of no use in diabetes control and in reducing the risk of diabetes in future. Many researchers used higher doses than normal dietary intake of vitamin C and proved that higher doses will be needed for glycemic control [22, 23]. In our study, nausea and abdominal discomfort were reported in one patient in placebo group while two patients in vitamin C group. These patients were treated by the physician in medicine OPD. All the patients responded to symptomatic treatment and continued the study. No serious adverse effects were reported in the study.

### 4.2. Limitations

Though the study sample size is small and duration is short, the value of results of the present study cannot be taken away. However, studies with a larger sample size and longer follow-up period together with measurement of other related antioxidant levels may yield more meaningful data on the role of the antioxidant system in the clinical course of type 2 DM. There is a need for increased number of follow-up visits to make this study more robust, broad-based and representative of the Indian population. Further studies on complicated and uncomplicated type 2 DM are required to elucidate exact role of vitamin C supplementation in type 2 DM.

### 4.3. Conclusion

Treatment with vitamin C with metformin was well tolerated and devoid of any side effects. The absence of any substantial side effects, cheaper cost, improvement in FBS, PPBS, and HBA1c, and the fact that plasma ascorbic acid levels are decreased in DM and increased after oral supplementation make it a particularly attractive therapeutic adjuvant in the treatment of type 2 DM.

## Figures and Tables

**Table 1:** Demographic characteristics of patients with type 2 diabetes mellitus.

Variables	Age (years)	Sex	
		Male	Female
Group A	48.33 ± 1.39	15	18
Group B	45.88 ± 1.42	13	20
*P* or *χ* ^2^	*P* = 0.2219, d.f = 64	*χ* ^2^ = 0.2481, d.f = 1	
*P* ^a^	>0.05	>0.05	

Values are given as Mean ± SEM, *n* = 33 in each group, Group A: vitamin C-treated group, Group B: placebo-treated group, ^a^ *P* values are ascertained by unpaired *t*-test or *χ* ^2^ test.

**Table 2:** Effect of metformin with placebo on FBG, PPBG, HbA1c, and plasma AA in patients with type 2 diabetes mellitus after 12 weeks of treatment.

Parameter	Before treatment	After treatment	*P* (paired)
FBG	160.75 ± 2.60	155.33 ± 2.31	<0.05
PPBG	218.51 ± 3.53	211.57 ± 2.88	<0.05
HbA1c	8.18 ± 0.12	8.01 ± 0.11	>0.05
Plasma AA	0.24 ± 0.006	0.27 ± 0.01	>0.05

*n* = 33 in number, FBG: fasting blood glucose, PPBG: postmeal blood glucose, HbA1c: glycosylated hemoglobin, plasma AA: plasma ascorbic acid. FBG, PPBG, and plasma AA are measured in mg/dL.

**Table 3:** Effect of metformin with vitamin C on FBG, PPBG, HbA1c and plasma AA in patients with type 2 diabetes mellitus after 12 weeks of treatment.

Parameter	Before treatment	After treatment	*P* (paired)
FBG	157.63 ± 3.13	141.18 ± 3.81	<0.01
PPBG	222.24 ± 3.16	206.69 ± 3.31	<0.05
HbA1c	8.26 ± 0.09	7.80 ± 0.08	<0.01
Plasma AA	0.26 ± 0.008	0.45 ± 0.01	<0.001

*n* = 33 in number, FBG: fasting blood glucose, PPBG: postmeal blood glucose, HbA1c: glycosylated hemoglobin, plasma AA: plasma ascorbic acid. FBG, PPBG, and plasma AA are measured in mg/dL.

**Table 4:** Comparison of effects of metformin with vitamin C and in combination with placebo on FBG, PPBG, HbA1c, and plasma AA at 12 weeks in patients with type 2 diabetes mellitus taking into account the change from baseline.

Parameter	Change from baseline at 12 weeks		*P* (unpaired)
	Group A	Group B	
FBG	−16.45 ± 3.80	−5.42 ± 2.65	<0.05
PPBG	−15.54 ± 2.42	−6.93 ± 2.99	<0.05
HbA1c	−0.451 ± 0.07	−0.169 ± 0.10	<0.05
Plasma AA	0.19 ± 0.01	0.02 ± 0.01	<0.001

*n* = 33 in each group, Group A: vitamin C-treated group, Group B: placebo-treated group, FBG: fasting blood glucose, PPBG: postmeal blood glucose, HbA1c: glycosylated hemoglobin, plasma AA: plasma ascorbic acid. FBG, PPBG, and plasma AA are measured in mg/dL.
